# The role of serum β-trophin and endostatin in patients with polycystic ovary syndrome: Are they correlated?

**DOI:** 10.1186/s12905-021-01198-4

**Published:** 2021-03-11

**Authors:** Wei Gong, Aikmu Bilixzi, Xinmei Wang, Yanli Lu, Li Wan, Lu Han

**Affiliations:** Department of Gynecology, The Fourth Affiliated Hospital of Xinjiang Medical University, No. 116 Huanghe Road, Urumqi, 214000 Xinjiang China

**Keywords:** β-Trophin, Endostatin, Polycystic ovary syndrome, Insulin resistance, Metabolism

## Abstract

**Background:**

It’s necessary to investigate the serum β-trophin and endostatin (ES) level and its influencing factors in patients with newly diagnosed polycystic ovary syndrome (PCOS).

**Methods:**

Newly diagnosed PCOS patients treated in our hospital were selected, and healthy women who took physical examination during the same period as healthy controls. We detected and compared the related serum indicators between two groups, Pearson correlation were conducted to identify the factors associated with β-trophin and ES, and the influencing factors of β-trophin and ES were analyzed by logistic regression.

**Results:**

A total of 62 PCOS patients and 65 healthy controls were included. The BMI, WHI, LH, FSH, TT, FAI, FBG, FINS, HOMA-IR, TC, TG, LDL, ES in PCOS patients were significantly higher than that of healthy controls, while the SHBG and HDL in PCOS patients were significantly lower than that of healthy controls (all *p* < 0.05). β-trophin was closely associated with BMI (r = 0.427), WHR (r = 0.504), FBG (r = 0.385), TG (r = 0.405) and LDL (r = 0.302, all *p* < 0.05), and ES was closely associated with BMI (r = 0.358), WHR (r = 0.421), FBG (r = 0.343), TC (r = 0.319), TG (r = 0.404, all *p* < 0.05). TG, BMI, WHR and FBG were the main factors affecting the serum β-trophin levels (all *p* < 0.05). FBG, TC and BMI were the main factors affecting the serum ES levels (all *p* < 0.05). The TG, β-trophin, ES level in PCOS patients with insulin resistance (IR) were significantly higher than that of those without IR (all *p* < 0.05).

**Conclusion:**

Increased β-trophin is closely associated with increased ES in patients with PCOS, which may be the useful indicators for the management of PCOS.

## Background

Polycystic ovary syndrome (PCOS) is one of the main causes of female infertility, with an incidence of about 6–12% in women of childbearing age [1, 2]. In recent years, with the improvement of living conditions, and the changes in the living environment of human beings, the onset of PCOS has increased significantly [3]. PCOS mainly manifests as imbalance of hypothalamic pituitary ovarian axis and metabolic disorders throughout the body. Its typical clinical features are irregular menstruation, chronic anovulation, obesity, hairiness, androgenemia, and some patients may be accompanied by metabolic abnormalities [4]. The tissue-specific Myo-inositol (MI) and D-chiro-inositol (DCI) ratio is modulated by insulin through aromatase and is altered in insulin resistance (IR), with reduced epimerization of MI to DCI in insulin-sensitive tissues [5]. It’s been reported that it has been dramatically reduced by insulin-stimulated epimerase in hyperinsulinemic women with PCOS, yet the potential mechanisms remain unclear [6, 7]. Therefore, in-depth research on the pathogenesis of PCOS is of great significance for improving the prognosis and quality of life of these patients.

Although the pathogenesis of PCOS is still not completely clear, IR and its compensatory hyperinsulinemia are one of its pathogenesis, and it’s been reported that 70% PCOS patients have the symptoms of IR [8, 9]. β-trophin, which is secreted by the liver and adipose tissue, has been found that plays a dual role in regulating lipid metabolism and improving glucose tolerance [10]. Previous studies [11, 12] have shown that serum β-trophin levels are higher in patients with IR-related metabolic diseases (diabetes, obesity, etc.), yet the role of β-trophin in PCOS needs further investigation. In recent years, studies [13, 14] have shown that ovarian angiogenesis and interstitial blood flow increase in patients with PCOS, so angiogenesis disorders may be one of the main reasons for the occurrence and development of PCOS. Endostatin (ES) is an angiogenesis inhibitor, which can inhibit the proliferation of new blood vessels [15]. Previous studies [16, 17] have shown that the levels of ES in patients with PCOS are abnormally elevated, but the specific mechanism needs further research. Therefore, we aimed to analyze the role of β-trophin and ES in patients with PCOS, to provide the insights and evidences into the treatment of PCOS.

## Methods

### Ethical consideration

The ethical approvals had been obtained from the ethical committee of The Fourth Affiliated Hospital of Xinjiang Medical University (2018120609-1c), and the written informed consents had been obtained from all the included participants. All the subjects were fully informed and volunteered to participate in the study. We aimed to conduct and report this study in comply with the Strengthening the Reporting of Observational Studies in Epidemiology (STROBE) Statement.

### Participants

Newly diagnosed PCOS patients treated in our hospital during December 2018 to February 2020 were selected as the study subjects. The diagnosis of PCOS was made according to the Rotterdam's criteria [18, 19]. The inclusion criteria for included patients were: (1) the age was ≥ 18 years and ≤ 35 years; (2) thin ovulation or anovulation; (3) clinical and biochemical manifestations of high androgens (including facial, prothoracic, and back area etc. for three consecutive months with multiple acne, hairy, seborrheic dermatitis, Total testosterone or androstenedione is higher than the normal reference value in the laboratory test); (4) polycystic changes in the ovaries are detected by ultrasound (the number of follicles with a diameter of 2–9 mm in one or both ovaries ≥ 12 and/or ovarian volume ≥ 10 mL). The exclusion criteria were as following: exclusion criteria: (1) patient complicated other diseases that cause elevated androgens, such as congenital adrenal hyperplasia, Cushing syndrome. And other diseases that cause ovulation disorders, such as hyperprolactinemia, abnormal thyroid function, premature ovarian failure, and pituitary or hypothalamic amenorrhea; (2) presence of moderate and severe hepatorenal dysfunction, or autoimmune disease; (3) presence of acute and chronic infectious diseases; (4) those who took hormones and other drugs that affect glucose and lipid metabolism in nearly three months.

Meanwhile, we selected healthy women who took physical examination in our hospital during the same period as healthy controls. The inclusion criteria were: (1) the menstrual cycle was regular and normal (28–35 days); (2) no clinical and biochemical manifestations of high androgen; (3) no polycystic ovarian changes were found under ultrasound detection.

### Data collection

The personal information such as the date of birth, menarche age, menstrual cycle, the time of menstruation disorder began, and the beginning time of hair change. At the same time, we measured the height (m), body weight (kg), and waist and hip circumference (cm). We calculated the body mass index (BMI) and waist to hip (WHR), and WHR = waist/hip circumference.

### Biochemical analysis

We collected 5 ml blood from the elbow vein on an empty stomach in the early morning (at least 8 h of fasting) from all participants. All blood samples were kept at room temperature for 30 min, and then centrifuged at 4000 r/min for 10 min. Then the serum was collected and stored in a refrigerator at − 80 °C. Following indicators were analyzed in our laboratory: fasting blood glucose (FBG), fasting insulin (FINS), luteinizing hormone (LH), follicle stimulating hormone (FSH), total testostrone (TT), androstendion (AND), sex hormone-binding globulin (SHBG), total cholesterol (TC), triglyceride (TG), high-density lipoprotein (high-ddensity lipoprotein (HDL), low-density lipoprotein (HDL). Besides, we calculated the homeostatic model assessment insulin resistance (HOMA-IR): HOMA-IR = (FBG × FINS)/22.5. Free testosterone index (FAI) = total testosterone (TT)/SHBG × 100.

Enzyme-linked immunosorbent assays (ELISA) kits (Shengmin, Hangzhou) were used to determine the serum β-trophin and ES level. All the operating procedures were performed strictly in accordance with the instructions.

### Statistical methods

This present study used SPSS 23. 0 statistical software for data analysis. The data conforms to the normal distribution and the variance is expressed by ${\overline{\chi }} \pm {\text{s}}$, otherwise it was expressed by M (P25, P75) and compared by log conversion to a normal distribution. T tests were conducted for comparing two independent samples. The relationship between variables was analyzed by Pearson correlation, and the influencing factors of β-trophin and ES were analyzed by logistic regression. The differences were considered as statistically significant if the *p* < 0.05.

## Results

### The characteristics of included participants

A total of 62 PCOS patients and 65 healthy controls were included in this present study. As Table 1 presented, there wasn’t any significant difference on the age between PCOS patients and healthy controls (*p* = 0.189). The BMI, WHI, LH, FSH, TT, FAI, FBG, FINS, HOMA-IR, TC, TG, LDL, ES in PCOS patients were significantly higher than that of healthy controls, while the SHBG and HDL in PCOS patients were significantly lower than that of healthy controls (all *p* < 0.05).

**Table 1:** The characteristics of included participants

Items	PCOS group (n = 62)	Control group (65)	t	*p*
Age	25.35 ± 3.58	25.11 ± 3.86	− 1.032	0.189
BMI (kg/m^2^)	24.28 ± 3.17	21.32 ± 3.29	4.205	0.018
WHR	0.90 ± 0.06	0.83 ± 0.05	1.127	0.011
LH (mIU/L)	9.03 ± 1.16	6.15 ± 1.12	1.184	0.009
FSH (IU/L)	5.96 ± 2.01	9.29 ± 2.15	1.202	0.013
TT (nmol/L)	2.28 ± 0.84	1.82 ± 0.71	1.047	0.025
SHBG (nmol/L)	32.83 ± 9.25	49.15 ± 8.19	− 12.184	0.015
FAI	9.70 ± 2.14	4.52 ± 1.18	2.105	0.029
AND (ng/mL)	3.94 ± 1.01	2.50 ± 0.96	1.146	0.012
FBG (mmol/L)	5.33 ± 1.13	4.66 ± 1.15	− 2.208	0.035
FINS (uIU/L)	23.29 ± 6.28	18.14 ± 2.24	2.995	0.000
HOMA-IR	3.92 ± 1.14	1.91 ± 0.97	1.028	0.007
TC (mmol/L)	4.86 ± 1.65	3.64 ± 1.90	1.136	0.015
TG (mmol/L)	1.43 ± 0.84	0.91 ± 0.11	1.177	0.023
HDL (mmol/L)	1.28 ± 0.22	1.32 ± 0.18	2.042	0.048
LDL (mmol/L)	2.83 ± 0.82	2.20 ± 0.74	1.192	0.017
β-trophin (pg/mL)	145.29 ± 30.22	99.30 ± 18.15	6.695	0.000
ES (μg/L)	313.09 ± 33.15	156.26 ± 28.85	20.150	0.000

### The correlation analysis

As Table 2 presented, β-trophin was closely associated with BMI (r = 0.427), WHR (r = 0.504), FBG (r = 0.385), TG (r = 0.405) and LDL (r = 0.302, all *p* < 0.05), and ES was closely associated with BMI (r = 0.358), WHR (r = 0.421), FBG (r = 0.343), TC (r = 0.319), TG (r = 0.404, all *p* < 0.05).

**Table 2:** The correlation analysis among β-trophin, ES and other related indicators

Items	β-Trophin		ES	
	R	*p*	r	*p*
Age	0.104	0.181	0.280	0.109
BMI	0.427	0.009	0.358	0.015
WHR	0.504	0.018	0.421	0.033
LH	0.118	0.094	0.038	0.299
FSH	0.206	0.059	0.332	0.095
TT	0.114	0.072	0.024	0.081
SHBG	0.121	0.090	0.137	0.075
FAI	0.299	0.207	0.260	0.130
AND	0.087	0.113	0.109	0.214
FBG	0.385	0.004	0.343	0.028
FINS	0.094	0.172	0.174	0.089
HOMA-IR	0.108	0.089	0.206	0.057
TC	0.142	0.091	0.319	0.006
TG	0.405	0.011	0.404	0.014
HDL	0.184	0.082	0.192	0.097
LDL	0.302	0.042	0.131	0.082

### Logistic regression analysis

Multivariate logistic regression analysis was performed with serum β-trophin as the dependent variable and BMI, WHR, FBG, TG and LDL as independent variables. The results showed that TG, BMI, WHR and FBG were the main factors affecting the serum β-trophin levels (all *p* < 0.05, Table 3).

**Table 3:** Logistic regression analysis on the factors related to β-trophin

Factors	β	S${\overline{\text{x}}}$	OR	95% CI	*p*	Rank
TG	0.88	0.27	4.37	1.14–9.54	0.012	1
BMI	0.93	0.30	5.32	1.25–10.80	0.030	2
WHR	1.01	0.46	4.80	1.36–9.85	0.041	3
FBG	0.92	0.38	5.66	2.27–13.46	0.028	4

Multivariate logistic regression analysis was performed with ES as the dependent variable and BMI, WHR, FBG, TC and TG as independent variables. The results showed that FBG, TC and BMI were the main factors affecting the serum ES levels (all *p* < 0.05, Table 4).

**Table 4:** Logistic regression analysis on the factors related to ES

Factors	β	S${\overline{\text{x}}}$	OR	95% CI	*p*	Rank
FBG	1.09	0.08	1.11	0.95–1.39	0.009	1
TC	0.22	0.15	1.64	1.34–1.98	0.016	2
BMI	1.19	0.30	1.16	0.96 ~ 1.28	0.029	3

### The comparison on the indicators of PCOS patients with and without IR

According to the HOMA-IR, the PCOS patients were divided into IR group (n = 38, HOMA-IR ≥ 2.69) and a non-IR group (n = 28, HOMA-IR < 2.69), we have checked and adjusted the BMI and WHR for IR comparison. As Table 5 presented, the TG, β-trophin, ES level in PCOS patients with IR were significantly higher than that of those without IR (all *p* < 0.05); There were no significant differences in age, BMI, WHI, LH, FSH,TT,FAI, SHBG, AND, FBG,FINS, HOMA-IR,TC,HDL, LDL between two groups (all *p* > 0.05, Table 5).

**Table 5:** The comparison of collected indicators in the PCOS patients with and without insulin resistance (IR)

Items	IR patients (n = 38)	No-IR patients (n = 24)	t	*p*
Age	25.34 ± 3.28	25.40 ± 3.17	1.214	0.227
BMI (kg/m^2^)	24.49 ± 2.24	24.15 ± 2.19	3.094	0.024
WHR	0.91 ± 0.09	0.90 ± 0.03	1.248	0.140
LH (mIU/L)	9.10 ± 1.29	9.01 ± 1.25	1.096	0.226
FSH (IU/L)	5.98 ± 2.11	5.96 ± 2.04	1.344	0.093
TT (nmol/L)	2.29 ± 0.39	2.27 ± 0.35	1.038	0.240
SHBG (nmol/L)	32.94 ± 9.44	31.99 ± 8.97	3.146	0.054
FAI	9.73 ± 2.33	9.69 ± 3.05	3.293	0.091
AND (ng/mL)	3.95 ± 1.14	3.82 ± 0.92	1.243	0.225
FBG (mmol/L)	5.34 ± 1.24	5.28 ± 1.26	1.135	0.073
FINS (uIU/L)	24.13 ± 3.22	23.94 ± 2.85	2.904	0.216
HOMA-IR	2.93 ± 1.25	2.67 ± 0.99	1.811	0.071
TC (mmol/L)	4.90 ± 1.85	4.87 ± 1.32	1.203	0.089
TG (mmol/L)	1.46 ± 0.49	1.39 ± 0.38	1.285	0.046
HDL (mmol/L)	1.29 ± 0.23	1.31 ± 0.21	1.146	0.142
LDL (mmol/L)	2.85 ± 0.83	2.87 ± 0.73	1.123	0.317
β-trophin	151.23 ± 32.09	141.29 ± 29.05	5.352	0.003
ES (μg/L)	319.03 ± 32.88	310.97 ± 24.29	18.135	0.015

## Discussion

Previous studies [20, 21] have pointed out that the incidence of PCOS in women of childbearing age ranges from 10 to 15%, and it remains an increasing trend. Angiogenesis is the basis for the growth of various tissues and organs in the body, but abnormal proliferation of blood vessels can cause diseases such as tumors and cysts in the body [22]. The main motive force and nutrients of vascular hyperplasia come from the blood supply [23]. When the blood supply increases, the nutrients absorbed by the new blood vessels will increase, which will accelerate the proliferation of new blood vessels. β-trophin is one of the most critical factors regulating angiogenesis, which can promote the proliferation of vascular endothelial cells and regulate vascular permeability [24]. Studies [25, 26] have shown that the abnormal expression, secretion and release of β-trophin in the ovaries may be one of the pathogenesis of PCOS. ES can selectively affect the vascular endothelial cells to inhibit the growth of endothelial cells and inhibit the formation of new blood vessels [27]. Related studies [28–30] have shown that in the serum of patients with ovarian cancer, endometrial cancer, breast cancer, hepatocellular carcinoma and other tumors, the β-trophin and ES concentrations have increased significantly. However, there are very few systematic reports to date on the correlation between β-trophin, ES and PCOS. In this context, we conducted this present study to identify the role of serum β-trophin, ES expression levels in the development of PCOS, the results have indicated that both serum β-trophin, ES are closely associated with the progress of PCOS, and they are highly involved in the process of IR, both β-trophin and ES may be sensitive indicators for the development of PCOS, and they may be the effective targets for the treatment of PCOS.

The cross-talk between altered metabolic and hormonal homeostasis in PCOS women must be considered. MI and DCI have been classified as insulin-sensitizers and seem to adequately counteract several InsR-related metabolic alterations with a safe nutraceutical profile [31]. It’s been reported that DCI-phosphoglycan and MI-phosphoglycan control key enzymes were involved in glucose and lipid metabolism [32, 33]. β-trophin is a highly effective and specific pro-angiogenic factor, which acts through tyrosine kinase receptors to promote increased vascular permeability, extracellular matrix degeneration, and vascular endothelial cell migration [34, 35]. In addition, elevated levels of androgen in patients with PCOS also lead to increased levels of β-trophin secreted by ovarian cells, which in turn exacerbates vascular proliferation in these patients [36]. ES is a highly effective and specific angiogenesis inhibitor. It mainly inhibits the proliferation of vascular endothelium in PCOS patients by promoting apoptosis of endothelial cells, thereby effectively inhibiting the formation of new blood vessels in PCOS patients [37]. Interesting, this present study has also found that the levels of β-trophin and ES in the IR group were higher than those in the non-IR group, suggesting that changes in serum β-trophin and ES levels are closely related to insulin resistance in patients with PCOS. It can be explained that the insulin can aggravate the activity of vascular endothelial cells and activate the renin–angiotensin–aldosterone system, resulting in elevated serum β-trophin and ES levels in patients with PCOS.

Previous studies [38, 39] have shown that β-trophin is mainly expressed by liver and adipose tissue in mice and is mainly secreted by liver in humans. Animal experiments [40, 41] have confirmed that in mice with betatrophin overexpression, the blood TG levels have increased significantly. While in β-trophin knockout mice, the blood TG level is decreased accordingly, and lipase activity increased. It is reported that β-trophin may regulate lipid metabolism by inhibiting lipase activity [42]. It is speculated that it may be involved in lipid metabolism disorders in patients with PCOS. The results of this study have showed that the serum β-trophin of PCOS patients is positively correlated with TG, and TG is an independent influencing factor of β-trophin, which is consistent with previous findings [43, 44].

Several limitations in this present study should be concerned. Firstly, we selected newly diagnosed PCOS patients treated in our hospital during December 2018 to February 2020 as targeted population, we did not perform sample size calculation, the sample size was rather small, it might fail to power enough to detect the potential associations. Secondly, we only detected the related indicators in newly diagnosed patients at the very first begning, and the dynamic changes of serum β-trophin and ES cannot be reflected. Besides, the molecular ratio of ES vs. β-trophin may be more helpful to show that if this parallel rise is comparable with controls or higher in either PCOS group, due to limited data, we cannot perform analysis on this issue. In the future, the molecular ratio of ES vs. β-trophin patients at different stages of the disease should be detected to further explore its relationship with PCOS. However, the advantage of this study is that the included PCOS patients are all newly diagnosed patients without serious glucose and lipid metabolism disorders, which may avoid the effect of drugs on β-trophin and EG, so it may more accurately reflect the related indicators of PCOS patients.

## Conclusions

In conclusion, the serum β-trophin and EG level in PCOS patients are higher than that of healthy people, both β-trophin and EG are closely related to PCOS. Furthermore, the role of β-trophin and EG may be highly associated with the glucose and lipid metabolism. Therefore, the detection of β-trophin and EG in patients' serum can be used to make the diagnostic and prognostic evaluation of PCOS. Further investigations in the future are needed to elucidate the potential mechanisms.

## Data Availability

Not applicable.

## Abbreviations

ES: Endostatin
PCOS: Polycystic ovary syndrome
MI: Myo-inositol
DCI: D-chiro-inositol
STROBE: Strengthening the Reporting of Observational Studies in Epidemiology
IR: Insulin resistance
FBG: Fasting blood glucose
FINS: Fasting insulin
LH: Luteinizing hormone
FSH: Follicle stimulating hormone
TT: Total testostrone
AND: Androstendion
SHBG: Sex hormone-binding globulin
TC: Total cholesterol
TG: Triglyceride
HDL: High-density lipoprotein
LDL: Low-density lipoprotein
HOMA-IR: Homeostatic model assessment insulin resistance
FAI: Free testosterone index
